# Chronic Alcoholism and Propofol Demand: The Impact of Alcohol Tolerance in Painless Gastrointestinal Endoscopy

**DOI:** 10.1002/jgh3.70326

**Published:** 2025-12-16

**Authors:** Xin Wang, Yue Shi, Hang Yang, Tianzhu Tao, Jun Ji

**Affiliations:** ^1^ Department of Anesthesiology Air Force Medical Center Beijing China; ^2^ Department of Anesthesiology China‐Japan Friendship Hospital, Chinese Academy of Medical Sciences and Peking Union Medical College Beijing China

**Keywords:** alcohol tolerance, anesthesia, closed‐loop target concentration infusion, endoscopy, propofol

## Abstract

**Background and Aim:**

Existing research suggests that chronic alcohol consumption may increase propofol requirements during anesthesia, though findings remain inconsistent. This study investigated the influence of chronic ethanol intake and alcoholic tolerance on propofol dosage in patients undergoing painless gastrointestinal endoscopy.

**Methods:**

One hundred male patients with habitual alcohol consumption were enrolled. Drinking behavior was assessed using the Alcohol Use Disorders Identification Test (AUDIT), and alcohol tolerance was also assessed by self‐reporting. Propofol was infused using a bispectral index (BIS)‐guided closed‐loop target‐controlled infusion system during induction and maintenance of anesthesia. The depth of anesthesia was controlled to maintain a BIS value of 60 ± 5, and the target plasma and effect‐site concentrations were recorded throughout the procedure.

**Results:**

Patients with high alcohol tolerance, but not hazardous drinking behavior, consumed higher propofol doses and exhibited higher effect‐site concentrations during anesthesia induction (BIS reach 60), maintenance (10 min post induction), and recovery (eye‐opening under stimuli). Both higher alcohol tolerance and hazardous drinking behavior were associated with shorter recovery times.

**Conclusions:**

Propofol requirements are increased in patients with high alcohol tolerance during painless gastrointestinal endoscopy. The mechanisms underlying this association, including potential pharmacodynamic adaptations, warrant further investigation.

## 1 Introduction

According to the World Health Organization (WHO), approximately 43% of the global adult population consumed alcohol in 2018 [1]. The harmful use of alcohol leads to a substantial burden of disease and carries significant social and economic consequences. Alcohol consumption is linked to an increased risk of mental and behavioral disorders, including alcohol dependence, as well as major noncommunicable diseases like liver cirrhosis, certain cancers, and cardiovascular diseases [2, 3, 4].

The question of whether alcohol consumption affects patients' tolerance to general anesthesia has long been a subject of debate. Previous studies have reported an increased induction dose of propofol in patients with chronic alcoholism [5, 6]. Conversely, Servin et al. found that total dose and predicted concentration of propofol were not affected by chronic alcoholism, and chronic alcoholism induces only mild changes in the pharmacokinetics of propofol [7]. More recently, a study in Chinese male patients showed that long‐term high‐risk drinking did not alter the effective dose of propofol required for successful insertion of gastroscope [8]. The discrepancies across these studies may be explained by the differences in population demographics, propofol administration methods, and outcome measurements.

Furthermore, compared to chronic alcohol consumption, alcohol tolerance appears to be another significant factor influencing the dosage of anesthetics, yet this issue has not been addressed in the literature. Thus, this study was designed to explore the potential correlation between hazardous drinking, alcohol tolerance, and propofol dose requirements during bispectral index (BIS) guided closed‐loop target‐controlled infusion in patients undergoing gastrointestinal endoscopy.

## 2 Methods

### 2.1 Study Design

This prospective observational cohort study aimed to enroll 100 patients with habitual alcohol consumption and was scheduled for gastrointestinal endoscopy. The protocol of the study was approved by the Ethics Committee Board of the Air Force Medical Center in Beijing, China (Approval No. 2023‐15‐S01) and was registered with the Chinese Clinical Trial Registry (Registration No. ChiCTR2300073694; registration date: July 19, 2023). Written informed consent was obtained from all participants.

### 2.2 Patients

Eligible patients were 25–55 years old with American Society of Anesthesiologists (ASA) physical status of I or II, and presented with normal hepatic and renal function. Exclusion criteria comprised: (1) neurological disorders such as acute stroke, Alzheimer's disease, history of neurosurgery, Parkinson's disease, and so forth; (2) severe vital organ dysfunction; (3) allergy to propofol; (4) past or present use of hypnotics, anxiolytics, or other central nervous system (CNS)‐acting agents except alcohol, and so forth; (5) suspicious difficult airway; (6) body mass index (BMI) > 30 or < 18.5 kg/m^2^.

These patients are divided into two cohorts: a hazardous/harmful drinking group (Group HD) and a nonhazardous drinking group (Group NHD) according to the alcohol use disorders identification test (AUDIT). AUDIT was developed by the WHO as a simple method to identify individuals who may have alcohol‐related issues. It consists of a series of questions focusing on various aspects of alcohol consumption patterns, drinking behaviors, and alcohol‐related consequences. A total score of 8 or more represents hazardous and harmful alcohol drinking [9].

Alcohol tolerance was assessed via a structured self‐report questionnaire (see in the Supporting Information). Participants provided a detailed history of alcohol‐induced intoxication, including the type, speed, and quantity of alcohol consumed. We use the intoxication dosage (minimal alcohol consumed within 2 h) divided by body weight as an assessment index for alcohol tolerance. Higher values indicate greater tolerance, reflecting the capacity to consume more alcohol before exhibiting typical signs of intoxication (e.g., emotional changes, impaired speech and coordination, behavioral changes, cognitive impairment, vomiting or confusion). Trained research personnel, not involved in clinical anesthesia or patient care, administered the questionnaire and obtained written informed consent.

### 2.3 Anesthesia and Monitoring

Without premedication, patients were monitored with noninvasive blood pressure, electrocardiogram, pulse oximetry (SpO_2_), BIS, and end‐tidal carbon dioxide (EtCO_2_). Propofol was delivered via a closed‐loop target‐controlled infusion (TCI) system (Marsh model, Silugao Medical Technology Co. Ltd., Beijing, China) which titrated the infusion rate using the BIS value as a feedback parameter [10]. Prior to initiation, patient demographics (age, sex, weight, and height) were entered into the system. In automatic mode, the system updated electroencephalographic data of patients every 5 s and determines the BIS error.

The target plasma concentration of propofol was initially set as 6.5 μg/mL, and was automatically adjusted to maintain a BIS value of 60 ± 5 throughout the procedure. Insertion of gastroscope was performed when the BIS value reached 60. If coughing occurred, 5 μg of sufentanil was administered intravenously. The target concentration of propofol (both plasma and effect‐site) was recorded at three time points: during induction, at maintenance (10 min after anesthesia onset) and upon recovery (defined as eye‐opening in response to verbal or tactile stimulation). The following parameters were documented: anesthesia time, recovery time, total propofol consumption, incidence of coughing, hypertension, hypotension, bradycardia, hypoxemia, and agitation.

### 2.4 Statistical Analysis

Normally distributed continuous variables were presented as mean ± standard deviation (SD), while non‐normally distributed variables were expressed as median with interquartile range (IQR). Categorical data were depicted as frequencies and percentages. Group differences in continuous variables were assessed using the independent samples *t*‐test or Mann–Whitney *U* test, and dichotomous variables were compared using the chi‐square test or Fisher's exact test, as appropriate. The relationship between hazardous drinking status, alcohol tolerance, and propofol concentration was assessed using Pearson correlation analysis. All statistical analyses were performed using Graphpad Prism software, version 6.0 (San Diego, CA, USA). A two‐tailed *p* value of less than 0.05 was considered statistically significant.

## 3 Results

### 3.1 Patient Inclusion and Basic Characteristics

Between July 1 and December 31, 2023, a total of 100 patients were enrolled in this study, with 50 patients in each of the NHD and HD groups. The demographic and clinical characteristics of both groups were illustrated in Table 1. Patients in the NHD group exhibited significantly shorter stature and lower body weight than those in the HD group; however, no significant intergroup difference was observed in BMI. No significant difference was observed in terms of other characteristics between the two groups.

**TABLE 1:** Baseline and clinical characteristics of patients.

Characteristics	Total (*n* = 100)	NHD (*n* = 50)	HD (*n* = 50)	*p*
Age (years)	45.8 ± 9.24	45.0 ± 9.24	46.6 ± 9.26	0.389
Body height (m)	1.743 ± 0.05	1.72 ± 0.05	1.74 ± 0.04	0.029*
Body weight (kg)	75.46 ± 7.56	73.54 ± 7.58	77.38 ± 7.11	0.010*
BMI	25.08 ± 2.23	24.75 ± 2.41	25.42 ± 1.99	0.133
Smoking history	59 (59)	33 (66)	26 (52)	0.222
Underlying conditions	37 (37)	19 (38)	18 (36)	0.999
CHD	1 (1)	0 (0)	1 (2)	0.999
Hypertension	33 (33)	16 (32)	17 (34)	0.999
Diabetes	9 (9)	7 (14)	2 (4)	0.159
Alcohol tolerance (g/kg)	0.24 ± 0.06	0.24 ± 0.05	0.25 ± 0.06	0.367
Anesthesia time (min)	18.13 ± 3.87	17.8 ± 3.28	18.50 ± 4.43	0.371
Recovery time (min)	4.89 ± 1.34	5.76 ± 1.29	4.88 ± 1.20	0.003*
Propofol consumption (mg/kg/min)	0.30 ± 0.06	0.30 ± 0.06	0.30 ± 0.069	0.990
Cough	8	2	6	0.268

*Note:* Data presented as *n* (%) or mean ± SD.

Abbreviations: BIS, bispectral index; BMI, body mass index; CHD, coronary heart disease; HD, hazardous and harmful drinkers; NHD, no hazardous drinkers.

* *p* < 0.05.

### 3.2 Hazardous Drinking Did Not Affect the Dosage of Propofol

**FIGURE 1:**
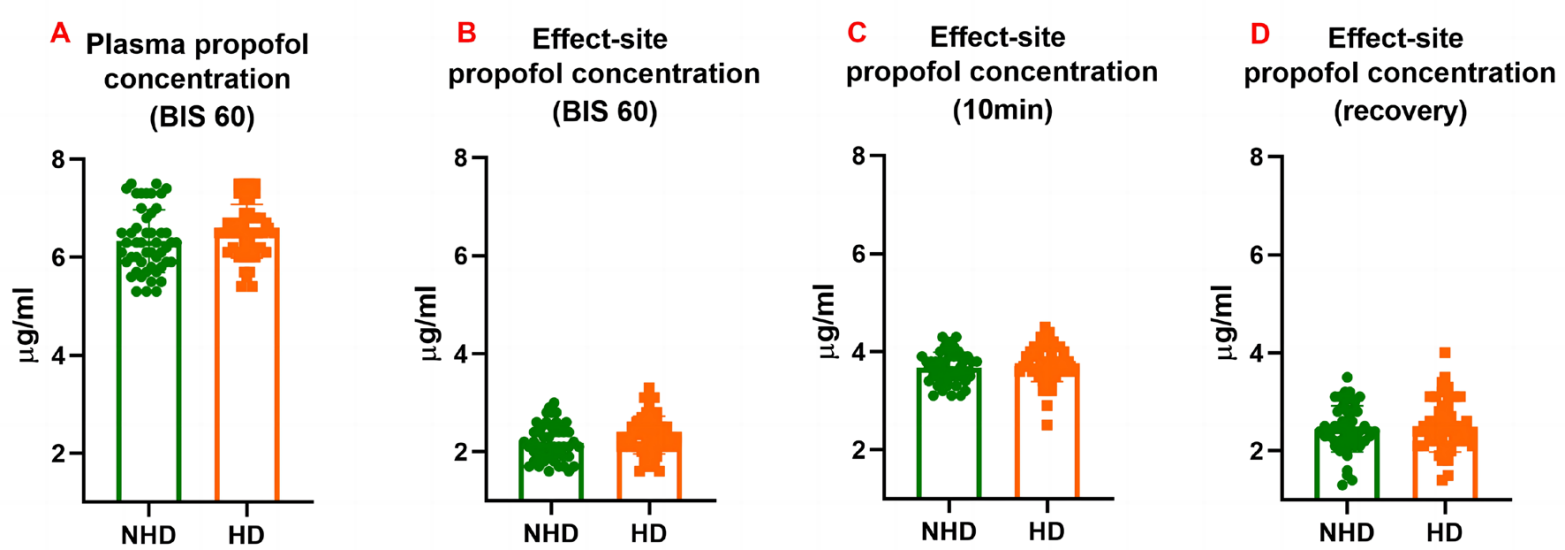
Propofol demand in patients with hazardous drinking (HD) and nonhazardous drinking (NHD). Target propofol concentrations were compared between NHD and HD groups. No significant difference was observed with respect to plasma propofol concentration (A) or effect‐site propofol concentration (B) when BIS reached 60, effect‐site concentration at 10 min post anesthesia induction (C), or recovery with eye‐opening under stimuli (D).

To facilitate a better comparison of intergroup propofol dosage, the obtained raw data were adjusted for body weight and anesthesia time. No significant difference was observed in the consumption of propofol between the HD and NHD groups. Similarly, no significant intergroup differences were found in plasma or effect‐site propofol concentrations when the BIS value reached 60. During both the maintenance and recovery phases of anesthesia, effect‐site propofol concentrations remained comparable between the HD and NHD groups (Figure 1). Recovery time, defined as the interval from cessation of propofol infusion to eye‐opening in response to verbal stimuli, was significantly shorter in the HD group compared to the NHD group (Table 1).

### 3.3 Correlation Between Propofol Dosage and Alcohol Tolerance

**FIGURE 2:**
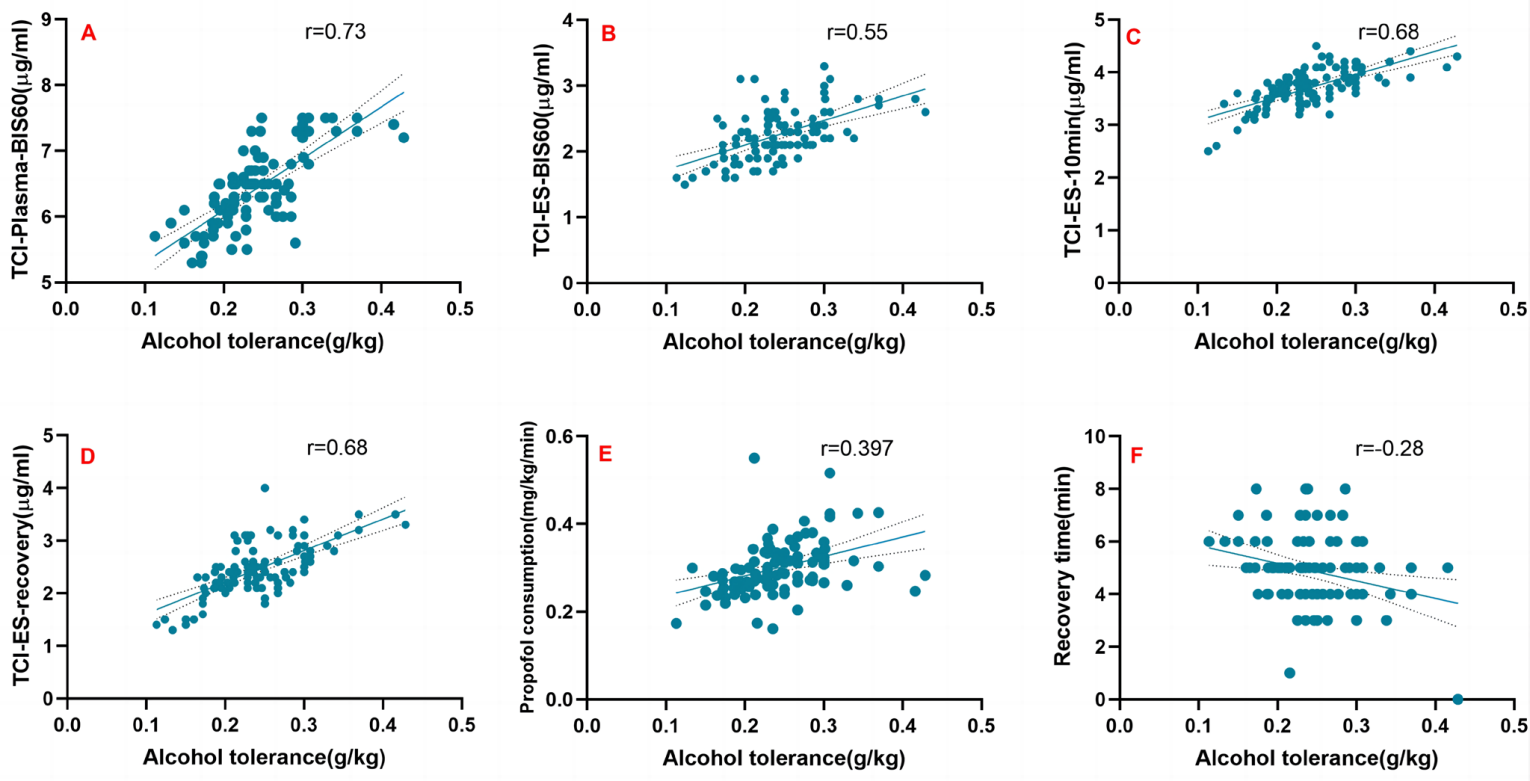
Correlation between alcohol tolerance and propofol demand in patients undergoing painless gastrointestinal endoscopy. Alcohol tolerance was positively correlated with target propofol concentration during BIS‐guided closed‐loop TCI infusion. (A) Plasma concentration when BIS reached 60; (B) effect‐site concentration when BIS reached 60; (C) effect‐site concentration at 10 min post induction; (D) effect‐site concentration at recovery with eye‐opening. Propofol consumption was increased in patients with high alcohol tolerance (E). Recovery time was decreased in patients with high alcohol tolerance (F).

Alcohol tolerance, quantified as the number of standard drinks adjusted for body weight, did not differ significantly between the HD and NHD groups. However, a significant positive correlation was observed between alcohol tolerance and propofol requirements. Patients with higher alcohol tolerance required greater total doses of propofol (*r* = 0.397, *p* < 0.001) and exhibited higher effect‐site and plasma concentrations during anesthesia induction (*r* = 0.55, *p* < 0.001; *r* = 0.73, *p* < 0.001, respectively), maintenance (*r* = 0.68, *p* < 0.001), and recovery (*r* = 0.68, *p* < 0.001). Correspondingly, we found that patients with high alcohol tolerance tend to have shorter recovery time (*r* = −0.28, *p* = 0.005) (Figure 2).

## 4 Discussion

Patients with chronic alcoholism frequently require surgery and general anesthesia. It is commonly accepted that, with normal hepatic and renal function, the dosage of anesthetic drugs should be appropriately increased for chronic alcoholics. Furthermore, patients with alcohol tolerance were likely to require higher anesthetic doses to achieve an equivalent depth of anesthesia. However, empirical evidence supporting a direct correlation between alcohol consumption and anesthetic requirements remains inconsistent. In this study, we investigated the association between drinking behavior and propofol requirements in patients undergoing gastrointestinal endoscopy with BIS‐guided closed‐loop target‐controlled infusion. The principal finding is that alcoholic tolerance, but not hazardous drinking behavior, is positively correlated with propofol dosage during both induction and maintenance of anesthesia. Additionally, patients with hazardous drinking or high alcohol tolerance exhibited shorter recovery times during propofol anesthesia.

Alcohol tolerance refers to the physiological adaptation to the effects of ethanol present in alcoholic beverages [11]. This encompasses direct tolerance, the rate of recovery from intoxication, and resistance to the onset of alcohol use disorder. Alcohol tolerance is influenced by numerous factors, including chronic alcoholism, body size, genetic variations in alcohol dehydrogenase activity, ethnicity, socioeconomic status, diet and drinking patterns [12, 13, 14]. To determine the correlation between alcohol tolerance and propofol requirements, we meticulously reviewed the patients' history of alcohol intoxication and corrected the alcohol dosage obtained by factoring in their body weight. Notably, in this cohort of habitual drinkers, we observed no significant correlation between hazardous drinking and the quantified level of alcohol tolerance. Specially, patients with high alcohol tolerance required significantly higher plasma and effect‐site concentrations of propofol to achieve the target BIS value of 60. This result suggests that the increased anesthetic requirement observed in alcohol‐tolerant individuals is unlikely to be explained solely by pharmacokinetic alterations.

The impact of alcohol on the CNS is closely linked to the γ‐aminobutyric acid (GABA) receptors, which represent a primary target through which propofol produces its anesthetic effects [15, 16]. GABA is the principal inhibitory neurotransmitter, regulating neuronal excitability by binding to GABA receptors on neurons. Alcohol potentiates the activity of these receptors, resulting in heightened neuronal inhibition and thus inducing sedative and relaxing effects. However, chronic alcohol exposure may lead to a diminished responsiveness of the CNS to GABA, necessitating greater alcohol consumption to achieve equivalent inhibitory effects [17, 18]. Supporting this, Hemby et al. demonstrated that the mRNA expression of β2 receptor in specific cortical regions of alcohol‐exposed primates [19]. Additionally, alcohol can influence other neurotransmitter systems, including glutamate and dopamine, which modulate mood, cognition, and behavior [20]. Therefore, alcohol's impact on the CNS is multifaceted, involving complex adaptations within GABAergic pathways and interactions with other neurotransmitter systems. These neurobiological adaptations likely contribute to the observed reduction in sensitivity to propofol in individuals with high alcohol tolerance.

Previous studies investigating the relationship between drinking behavior and propofol dosage have yielded inconsistent conclusions. Liang et al. reported that the induction dose requirements of propofol were increased in alcoholic patients anesthetized with propofol and remifentanil administered by target‐controlled infusion [6]. In contrast, Servin et al. found that the total propofol dose and the predicted and measured concentrations during all phases of anesthesia did not differ between the chronic alcoholic and control patients. Chronic alcoholism induced only mild changes in the pharmacokinetics of propofol [7]. More recently, Xue et al. reported no significant difference in the estimated ED_50_ of propofol required for successful insertion of gastroscope between long‐term high‐risk drinkers and nondrinkers among Chinese male patients [8]. These discrepancies may be attributable to several confounding factors, including variations in age, gender, definitions of alcoholism, depth of anesthesia, methods of propofol administration, and observed indicators.

To mitigate these confounders, the present study implemented several methodological refinements. The cohort was restricted to male patients aged 25–55 years, who received propofol sedation exclusively without the influence of other sedatives or analgesics. To standardize anesthetic depth and delivery, we employed a BIS‐guided closed‐loop target‐controlled infusion system, maintaining a BIS value of 60 ± 5, and utilized real‐time target concentrations as evaluation indicators. Additionally, we simultaneously analyzed the relationship between hazardous drinking, alcohol tolerance, and propofol consumption, both in induction, maintenance, and recovery. This approach allowed for a more comprehensive and accurate assessment of the relationship between drinking behavior and propofol dosage.

Although this study has yielded some valuable insights, several limitations should be acknowledged. First, we assessed alcohol tolerance through self‐reporting, yet some patients might not accurately recall their alcohol consumption before intoxication. Second, propofol requirements were evaluated using the target concentrations generated by the closed‐loop target‐controlled infusion system. Actual plasma propofol concentrations were not measured, limiting our ability to assess potential pharmacokinetic alterations directly. Finally, the closed‐loop target‐controlled infusion using the Marsh model demonstrated good stability during the maintenance phase of anesthesia; however, its accuracy during the induction phase still requires further clinical evidence.

In conclusion, the present study reported for the first time that alcoholic tolerance, but not hazardous drinking behavior, is positively correlated with increased propofol dose requirements during both induction and maintenance of anesthesia. Moreover, patients with either hazardous drinking or high alcohol tolerance exhibited shorter recovery times during propofol anesthesia. Alcohol tolerance alters the dosage of propofol required, and the potential underlying mechanism warrants further investigation.

## Funding

This work was supported by the PhD Booster Program of the Air Force Medical Center (2021ZT020 and 2022YXQN014).

## Ethics Statement

This study was approved by the Ethics Committee Board of the Air Force Medical Center (No. 2023‐15‐S01) and registered in the Chinese Clinical Trial Registry (No. ChiCTR2300073694; registered on July 19, 2023).

## Consent

The written informed consent was obtained from each patient.

## Conflicts of Interest

The authors declare no conflicts of interest.

## Supporting information


**Data S1:** jgh370326‐sup‐0001‐Supinfo.docx.

## Data Availability

The data that support the findings of this study are available on request from the corresponding author. The data are not publicly available due to privacy or ethical restrictions.
