# Supplementary material for: Chronic Alcoholism and Propofol Demand: The Impact of Alcohol Tolerance in Painless Gastrointestinal Endoscopy
Source: JGH Open. 2025 Dec 16;9(12):e70326. doi: 10.1002/jgh3.70326 (PMC12706821; doi:10.1002/jgh3.70326)
Supplement: Supplementary file 1 — Data S1: jgh370326‐sup‐0001‐Supinfo.docx. [file JGH3-9-e70326-s001.docx]

**Alcohol Use Disorders Identification Test (AUDIT)**

| **Name:** |  | **Sex:** |  | **Age:** |  |
| --- | --- | --- | --- | --- | --- |
| **Height:** |  | **Weight:** |  | **Smoking history:** | ___years  ___ cigarettes/day |
| **Past history:** | Hypertension□; Diabetes□;  Liver diseases□; Other diseases: | | | **Drinking history:** | ___ years |

***** How many drinks does it take to get you drunk?

(At least one of the following is met: 1. impaired consciousness; 2. euphoria, loss of self-control, reckless behavior; 3. depressed mood with few words)

Chinese liquor (38% concentration) ___liang or Chinese liquor (52% concentration) ___liang or 500ml beer _______ bottle (within 2 hours)

1. How often do you have a drink containing alcohol?

· Never □

· Monthly or less □

· 2-4 times a month □

· 2-3 times a week □

· 4 or more times a week □

2. How many standard drinks containing alcohol do you have on a typical day when drinking?

· 1 or 2 □

· 3 or 4 □

· 5 or 6 □

· 7 to 9 □

· 10 or more □

3. How often do you have six or more drinks on one occasion?

· Never □

· Less than monthly □

· Monthly □

· Weekly □

· Daily or almost daily □

4. During the past year, how often have you found that you were not able to stop drinking once you had started?

· Never □

· Less than monthly □

· Monthly □

· Weekly □

· Daily or almost daily □

5. During the past year, how often have you failed to do what was normally expected of you because of drinking?

· Never □

· Less than monthly □

· Monthly □

· Weekly □

· Daily or almost daily □

6. During the past year, how often have you needed a drink in the morning to get yourself going after a heavy drinking session?

· Never □

· Less than monthly □

· Monthly □

· Weekly □

· Daily or almost daily □

7. During the past year, how often have you had a feeling of guilt or remorse after drinking?

· Never □

· Less than monthly □

· Monthly □

· Weekly □

· Daily or almost daily □

8. During the past year, have you been unable to remember what happened the night before because you had been drinking?

· Never □

· Less than monthly □

· Monthly □

· Weekly □

· Daily or almost daily □

9. Have you or someone else been injured as a result of your drinking?

· No □

· Yes, but not in the past year □

· Yes, during the past year □

10. Has a relative or friend, doctor or other health worker been concerned about your drinking or suggested you cut down?

· No □

· Yes, but not in the past year □

· Yes, during the past year □

1 standard drink = 10 grams of pure alcohol

1 bottle of 750ml wine = 9 standard drinks

1 bottle of beer = 2 standard drinks

1 bottle of 750ml yellow wine or rice wine = 6 standard drinks

1 liang of Chinese liquor (52% concentration) = 2 standard drinks

1 liang of Chinese liquor (45% concentration) = 1.8 standard drinks

1 liang of Chinese liquor (38% concentration) = 1.5 standard drinks

Grams of alcohol consumed = milliliters of alcohol consumed * alcohol content * 0.8

Scoring the AUDIT:

Scores for each question range from 0 to 4, with the first response for each question (eg never) scoring 0, the second (eg less than monthly) scoring 1, the third (eg monthly) scoring 2, the fourth (eg weekly) scoring 3, and the last response (eg Daily or almost daily) scoring 4. For questions 9 and 10, which only have three responses, the scoring is 0, 2 and 4 (from left to right).

A score of 8 or more is associated with harmful or hazardous drinking, and is likely to indicate alcohol dependence.
